# A dataset for machine learning-based QSAR models establishment to screen beta-lactamase inhibitors using the FARM -BIOMOL chemical library

**DOI:** 10.1186/s13104-025-07159-6

**Published:** 2025-03-03

**Authors:** Thanet Pitakbut, Jennifer Munkert, Wenhui Xi, Yanjie Wei, Gregor Fuhrmann

**Affiliations:** 1 Department of Biology, Pharmaceutical Biology, Friedrich-Alexander-Universität Erlangen-Nürnberg, Staudtstr. 5, 91058 Erlangen, Germany; 2 FAU NeW– Research Center New Bioactive Compounds, Nikolaus-Fiebiger-Str. 10, 91058 Erlangen, Germany; 3 Shenzhen Key Laboratory of Intelligent Bioinformatics and Center for High-Performance Computing, Shenzhen Institute of Advanced Technology, Chinese Academy of Sciences, Shenzhen, 518055 China

**Keywords:** Machine learning, Random forest, Logistic regression, Molecular docking, AutoDock vina, DOCK6, Consensus docking, Physio-chemical descriptors, And QSAR

## Abstract

**Objectives:**

Beta-lactamase is a bacterial enzyme that deactivates beta-lactam antibiotics, and it is one of the leading causes of antibiotic resistance problems globally. In current drug discovery research, molecular simulation, like molecular docking, has been routinely integrated to virtually screen an enzyme inhibitory effect. However, a commonly known limitation of molecular docking is a low percent success rate. Previously, we reported a proof-of-concept of combining machine learning with a quantitative structure-activity relationship (QSAR) model that overcame this limitation (10.1186/s13065-024-01324-x). Here, we presented and navigated the dataset used in our previous report, including sixty trained models (thirty for random forest and another thirty for logistic regression).

**Data description:**

This data note has three essential parts. The first part is an in vitro beta-lactamase inhibitory screening of eighty-nine bioactive molecules. The second part consisted of three molecular docking approaches (AutoDock Vina, DOCK6, and consensus docking). The last part is machine learning integrated with QSAR models. Therefore, this data note is vital for further model development to increase performance.

## Objective

Beta-lactamase is a bacterial enzyme produced to resist beta-lactam antibiotic drugs [1], one of the three largest antibacterial classes commonly used to treat infection [2]. Therefore, beta-lactamase contributes to a current drug-resistant infection problem worldwide. Even though computational docking simulation offers a fast pace in the drug discovery process, it has a significant limitation: a low percentage success rate [3, 4]. To provide a prove-of-concept to overcome this limitation, this dataset was collected using an in-house chemical library, FARM-BIOMOL - *FAUPharmaceutical* biology -*BioactiveMolecules* (https://pharmbio-fau-erlangen.github.io/FARM-BIOMOL/), at the Division of Pharmaceutical Biology, Department of Biology, Faculty of Natural Sciences, Friedrich-Alexander-Universität Erlangen-Nürnberg, Erlangen, Germany [5]. Our previous work used this dataset to establish machine learning-based quantitative structure-activity relationship (QSAR) models (10.1186/s13065-024-01324-x) [6]. There are three main aims in this data note. The first aim is to share our biological experimental data with the public to advance anti-biotic-resistant drug discovery studies since there are some first-time reports of natural products against beta-lactamase. The second and third aims are to grant the public access to our optimized docking protocol for virtual screening against beta-lactamase and all sixty constructed machine learning models (thirty for random forest and another thirty for logistic regression).

## Data description

### Beta-lactamase inhibitory screening

In this data file (Data file 1, Table 1), eighty-nine biomolecules from FARM-BIOMOL [5] were tested against beta-lactamase using a standard in vitro colorimetric enzyme binding assay monitored by a microplate reader from our previous work. The basic principle of this assay is when there is no inhibitor in the test system, the free enzymes can freely convert substrates to products, leading to a change of color in the test system. On the other hand, when inhibitors are presented in the test system, there will be fewer free active enzymes, leading to a lower substrate-product conversion rate and altering the change of color in the test system. The color change here can be monitored using a microplate reader’s optical density (OD). Finally, each biomolecule tested system’s OD is normalized with the reference system’s OD (without an inhibitor) to calculate percent inhibition. In this data file, we provided a percent inhibition, standard deviation (SD), and standard error of mean (SEM) of eighty-nine tested biomolecules from the FARM-BIOMOL chemical library [5] with its simplified molecular input line entry system or SMILE data for a 1D chemical annotation.

### Docking simulation

As shown in Table 1, three files, data files 2 to 4, involve molecular docking simulation. The first data file (Data file 2) is for molecular docking obtained from AutoDock Vina or AD Vina [7]. The second data file (Data file 3) represents a docking result obtained from DOCK6 [8]. Finally, Data file 4 is an outcome of consensus docking, combining AD Vina and DOCK6 results. Even though docking simulations were conducted differently, the simulation’s principle was the same. Each software predicts a molecular binding score between a compound of interest’s optimized 3D chemical structure and the beta-lactamase binding site (an active site). Both data files (Data files 2 and 3) contained all 3D chemical structures of eighty-nine compounds and predicted binding score obtained from each software. They included a pre-processed 3D structure of beta-lactamase, eighty-nine compounds from FRAM-BIOMOL [5], a validated docking protocol, a virtual screening command script, and a result in a separate folder. Data file 4, consensus docking, was obtained by comparing and identifying AD Vina [7] and DOCK6 [8] results after sorting docking scores in percentile from each program. The 50% percentile was used as a cutoff threshold. The result of consensus was provided in two scoring systems. The first was a binary score (1 = consensus positive and 2 = consensus negative), and the second was a summary score combining AD Vina [7] and DOCK6 [8] binding scores.

### Machine learning-based QSAR model

We analyzed two machine learning algorithms: random forest (Data file 5) and logistic classification (Data file 6). Each algorithm generated thirty models, and the best model was defined by the highest accuracy and receiver operating characteristic area under the curve (ROC-AUC) scores. 1,875 physicochemical property descriptors were generated using PaDEL software [9], and a consensus binary score was used as an additional descriptor. Finally, a complete data set can be downloaded via Data Set 1 from Table 1.

**Table 1:** Overview of data files/data sets

Label	Name of data file/data set	File types (file extension)	Data repository and identifier (DOI or accession number)
Data file 1	Beta-lactmase_inhibitoryscreen.csv	.csv	Zenodo (10.5281/zenodo.13378954) [10]
Data file 2	Docking_vina.zip	.zip	Zenodo (10.5281/zenodo.13378954) [10]
Data file 3	Docking_dock6.zip	.zip	Zenodo (10.5281/zenodo.13378954) [10]
Data file 4	Docking_consensus.zip	.zip	Zenodo (10.5281/zenodo.13378954) [10]
Data file 5	RandomForest-QSAR.zip	.zip	Zenodo (10.5281/zenodo.13378954) [10]
Data file 6	Logistic-QSAR.zip	.zip	Zenodo (10.5281/zenodo.13378954) [10]
Data set 1	ThanetPi/ML-QSAR-Docking-Proof-of-Concept: v.1.0.2024	.zip	Zenodo (10.5281/zenodo.13378560) [11]

### Limitations

The main limitation of this dataset is its relatively small size. This limitation applies to broad aspects of this dataset, as shown below.

- From natural product chemistry, the chemical compounds in FARM-BIOMOL [5] only represent a fraction of the natural products class.
- From enzyme biology, only one category of beta-lactamase was tested (there are four in total) [12].
- From docking simulation, only two docking software were utilized.
- From machine learning and the QSAR model establishment, less specific physicochemical property descriptors were generated from an open-source program; this dataset only used non-engineer features and one machine learning algorithm (random forest).

Even if the proof-of-concept model was established and demonstrated an accepted performance, careful evaluation before using this data is required to avoid undesirable outcomes.

## Data Availability

The data described in this Data note can be freely and openly accessed on https://zenodo.org under 10.5281/zenodo.13378954. and 10.5281/zenodo.13378560. Please see Table 1 and references [10, 11] for details and links to the data.

## Abbreviations

FARM-BIOMOL: FAU Pharmaceutical Biology-Bioactive molecules chemical library
QSAR: Quantitative structure-activity relationship
OD: Optical density
SD: Standard deviation
SEM: Standard error of mean
AD Vina: AutoDock Vina
